# Induction of Size-Dependent Breakdown of Blood-Milk Barrier in Lactating Mice by TiO_2_ Nanoparticles

**DOI:** 10.1371/journal.pone.0122591

**Published:** 2015-04-07

**Authors:** Chengke Zhang, Shumei Zhai, Ling Wu, Yuhong Bai, Jianbo Jia, Yi Zhang, Bin Zhang, Bing Yan

**Affiliations:** School of Chemistry and Chemical Engineering, Shandong University, Jinan, China; Rutgers University, UNITED STATES

## Abstract

This study aims to investigate the potential nanotoxic effects of TiO_2_ nanoparticles (TNPs) to dams and pups during lactation period. TiO_2_ nanoparticles are accumulated in mammary glands of lactating mice after i.v. administration. This accumulation of TiO_2_ NP likely causes a ROS-induced disruption of tight junction of the blood-milk barrier as indicated by the loss of tight junction proteins and the shedding of alveolar epithelial cells. Compared to larger TNPs (50 nm), smaller ones (8 nm) exhibit a higher accumulation in mammary glands and are more potent in causing perturbations to blood-milk barrier. An alarming finding is that the smaller TNPs (8 nm) are transferred from dams to pups through breastfeeding, likely through the disrupted blood-milk barrier. However, during the lactation period, the nutrient quality of milk from dams and the early developmental landmarks of the pups are not affected by above perturbations.

## Introduction

TiO_2_ nanoparticles (TNPs) have been widely used in diverse areas. There have been more than 1,600 nanotechnology-based consumer products on the market [1]. Among them, about 197 products are based on TNPs. These products ranges from environmental remediation [2, 3], and cosmetics [4] to food additives [5], and nanomedicine [6], antibacterial materials [7, 8]. Applications of nanomaterials and nanotechnology have increased the environmental release and accumulation of nanoparticles and the human exposure to these materials [9, 10]. For example, TNPs released from exterior paint of urban buildings contaminate surface waters [10] and these nanoparticles cause toxicity in aquatic organisms [11].

Nano pollution to the environment and nanomedicine application of TNPs has raised concerns about the potential nanotoxic effects to humans, especially to vulnerable populations, such as lactating females. Breastfeeding, or lactation, is considered essential to growth [12], active and passive immunity [13], and cognitive and psychosocial development of newborns [14]. It has been recommended that exclusive breastfeeding should last for at least six months after a baby is born [13]. However, there have been indications that nanoparticles are present in rat milk via an unknown mechanism when lactating dams are exposed to nanoparticles [15, 16]. This finding indicates that nanoparticle exposure may pose dangers to both the mother and newborn during and after the lactation period.

Nanoparticles’ exposures through inhalation, digestion, and skin contact eventually lead to their absorption into the blood and their distribution to various organs [17]. Organs exhibit different sensitivities to nanoparticle perturbations. Physiological barriers are used to protect vulnerable organs or processes. Among these protections, the blood-milk barrier provides important protection for milk integrity and the health of pups. Nanoparticles induce the breakdown of some important physiological barriers [18–20]. Silica nanoparticles and TNPs induce blood-placental barrier damage [18]. Multiwalled carbon nanotubes [19] and gold nanoparticles [20] cross the blood–testis barrier and cause damage to the testis. Although nanoparticles were found in milk after dam’s exposure [15, 16], questions regarding whether and how nanoparticles compromise the blood-milk barrier remain unanswered.

In this investigation, we revealed the effects of TNPs on lactating dams and their pups during the lactation period after four intravenous (i.v.) administrations of TNPs of different sizes (8 nm and 50 nm). TNPs, especially the smaller ones, were observed to enter the mammary glands of dams, induce reactive oxygen species (ROS), and damage the integrity of the blood-milk barrier by causing shedding of alveolar epithelial cells and inducing gaps in the tight junction of the barrier. These effects allow the passage of TNPs into milk and subsequently to the pups’ gastrointestinal (GI) tract. At the applied doses and under our experimental conditions, the milk quality and the pups’ early developmental landmarks were not affected during the lactation period.

## Materials and Methods

Our experimental protocols complied with the NIH guidelines outlined in the Guide for the Care and Use of Laboratory Animals and were approved by the Committee on the Ethics on Animal Experimentation of Shandong University.

### TNP Characterization

Anatase-type TiO_2_ nanoparticles were purchased from Shanghai Jingchun Co., Ltd. (Shanghai, China). The crystal structure of TNP-8 and TNP-50 were determined by their X-ray diffraction patterns. The hydrodynamic size and the Zeta potential of the naked TNPs and NP-protein complexes were determined according to the method in the previous study [21]. Briefly, dynamic diameter of TNPs was measured by a particle size analyzer (Malvern Nano ZS90, Malvern, UK). TNPs were suspended in water or culture medium with 10% fetal bovine serum. For Zeta potentials analysis, TNPs were first diluted either in ultrapure water (18.2 MΩ) or in 10% FBS. After 24 h incubation, free and loose bound proteins were removed by centrifugation (13 000 rpm), and TNPs were washed and centrifuged for three times. The protein bound TNPs were then dispersed in water for their zeta potential measurement in a Malvern Nano ZS90 Zetasizer. Each material was tested three times.

### Mice and dosing

Timed pregnant CD-1 mice were purchased from Beijing Vital River Laboratories (Beijing, China).

The hour of the day at which a dam gave birth was designated as LD 0. The litter sizes were normalized to eight (four males and four females) on LD 2. At LD 2, the dams were randomly divided into eleven groups with seven per group (LD 10 endpoint group and LD 21 endpoint group). The dams targeted for LD 10 or LD 21 endpoint groups were dosed intravenously through the tail vein with PBS, TNP**-**8 (2, 6, and 8 mg/kg), and TNP**-**50 (2, 6, and 8 mg/kg) at LD 2, 4, 6, 8. Dams from a group for LD 10 end point were subcutaneously injected with CdCl_2_ (2 mg/kg) at LD 2, 4, 6, and 8.

### Ethics Statement

Our experimental protocols complied with the NIH guidelines outlined in the *Guide for the Care and Use of Laboratory Animals* and were approved by a local ethics committee.

### TNPs and TNPs injection solution preparation

The two TNPs’ purity which was given by the manufacturer was 99.81%. The TNP nanoparticles were dispersed in sterile PBS. Before injection, the solution was sonicated for 10 min, and vortexed for 1 min.

### Animal samples

#### Milk

Milk samples were obtained from dams at LD 10. Briefly, the entire litter was separated from the dam for approximately 2 h prior to milking. The dams were anesthetized with sodium pentobarbital (50 mg/kg) (Sigma, USA) by i.p. injection before milk collection. Then dams were given 2 IU of oxytocin (Sigma, USA) through i.p. injection. Milk collection from the right third thoracic mammary gland was initiated within 5 min after the administration of oxytocin. The ejected milk was collected by gentle aspiration[22]. The milk samples were frozen immediately and stored at -80°C.

#### Blood and organs

The dams of LD 10 and LD 21 endpoint groups were sacrificed after anesthesia using sodium pentobarbital at LD 10 and LD 21. Blood was added to heparin tube, plasma was harvested by centrifugation at 600 g for 15 min. The heart, liver, spleen, lungs, kidneys, uterus, and ovaries were collected and weighed. Mammary gland tissues for biodistribution analysis, oxidative stress analysis, RNA and protein extraction were placed on dry ice and stored at -80°C. To minimize individual variations, the left fourth and fifth inguinal mammary glands from each mouse were used for histopathological examination. The right fourth and fifth inguinal mammary glands from each mouse were used for RNA isolation, protein extraction, and analysis of oxidative stress. Left kidney, lung, and the left first, second, and third thoracic mammary glands were used for biodistribution study.

#### GI tract of Pups

After separation from the dams, the pups were weighed and sacrificed after anesthesia by cervical dislocation at LD 10. The GI tracts, including stomach and intestine from eight pups were excised and pooled for Ti content analysis. For LD 21 endpoint group, the pups were observed for hair growth beginning at LD 10 and for eye opening beginning at LD 13.

### Measurement of oxidative stress

The methods for homogenate preparation and analyses were carried out according to established procedures[23]. The livers and mammary gland tissues were homogenized in cold PBS using a tissue homogenizer (Bio-Gen PRO200 Homogenizer, Oxford, CT, USA) after weighing. The homogenates were centrifuged at 3,000 g for 15 min at 4°C and then the supernatant was collected. The malondialdehyde (MDA) and glutathione (GSH) levels in the supernatants were analyzed using MDA assay kit and GSH assay kit (Nanjing Jiancheng Bioengineering Institute, Jiangsu, China).

### Histopathological examination and counting of shed cells

Tissues from heart, liver, spleen, lungs, kidney and mammary gland were fixed in 10% formalin neutral buffer solution for 24 h. Paraffin-embedded sections (5 μm) of tissues were stained with hematoxylin and eosin. The pathologist was blinded to the identity and analysis of the pathology sections. To quantitatively analyze the numbers of cells shed into the alveolar lumen, seven mice per group were examined and approximately 100 images of alveoli per mouse were examined and counted.

### Protein extraction and Western blot analysis

Frozen mammary glands (0.1 g) were homogenized in 1.5 mL of whole-cell homogenization buffer (20 mM HEPES [pH 7.9]/ 20% (v/v) glycerol/ 250 mM KCl/ 2 mM MgCl_2_/ 0.2 mM EDTA/ 0.5 mM dithiothreitol/ 1 mM PMSF/ 0.1% Triton X-100/ 5 μL/mL of buffer). The extracts were centrifuged (50,000 g, 4°C, 15 min), and the supernatants were collected for subsequent analysis. The concentrations of the lysates were determined using a BCA assay (Bio-Rad, CA, USA). Equal volumes of protein (20 μg) were loaded onto 10% SDS/PAGE gels and then electrophoretically transferred onto a polyvinylidene difluoride membrane (Millipore, MA, USA) using a Mini Trans-Blot cell (Bio-Rad, CA, USA). The membranes were then incubated in 5% bovine serum albumin (BSA) (Sigma, USA) in Tris-buffered saline (TBS) with 0.1% Tween 20 and shaken for 1 h at room temperature, followed by incubation overnight at 4°C with primary antibodies against occludin (Epitomics, CA, USA), ZO-1 (Abcam, MA, USA) and GAPDH (ImmunoWay Biotechnology Company, Newark, DE, USA). The membranes were washed with TBS with 0.1% Tween 20 and probed with horseradish peroxidase-conjugated anti-rabbit or anti-mouse IgG (Bio-Rad, CA, USA). The proteins were detected with the Luminata Forte Western HRP substrate (Millipore, MA, USA) according to the manufacturer’s instructions.

### Titanium Content Analysis

The methods for titanium analysis were carried out according to established procedures [24, 25]. Tissue samples of approximately 0.2 g were weighed, digested, and analyzed for titanium content. The samples were digested in a microwave oven after the addition of 3 mL of ultrapure nitric acid and 1 mL of hydrogen peroxide. The digested solutions were diluted with ultrapure water. The titanium content was analyzed using inductively coupled plasma-mass spectrometry (ICP-MS, Agilent 7700, Santa Clara CA, USA). Indium (20 ng/mL) was used as an internal standard. The detection limit of titanium was 1.87 ng/L.

### Statistical Analysis

All of the statistical calculations were performed using SigmaPlot 12.0. The results are reported as the mean±s.d. of multiple independent determinations. The comparisons between the control and experimental groups and the differences within experimental groups at different doses were analyzed using one-way ANOVA followed by least-significant difference or Tukey’s tests. A P value of less than 0.05 was considered significant.

## Results and Discussion

### Accumulation of TNPs in major organs does not induce acute toxicity in lactating dams

**Fig 1:**
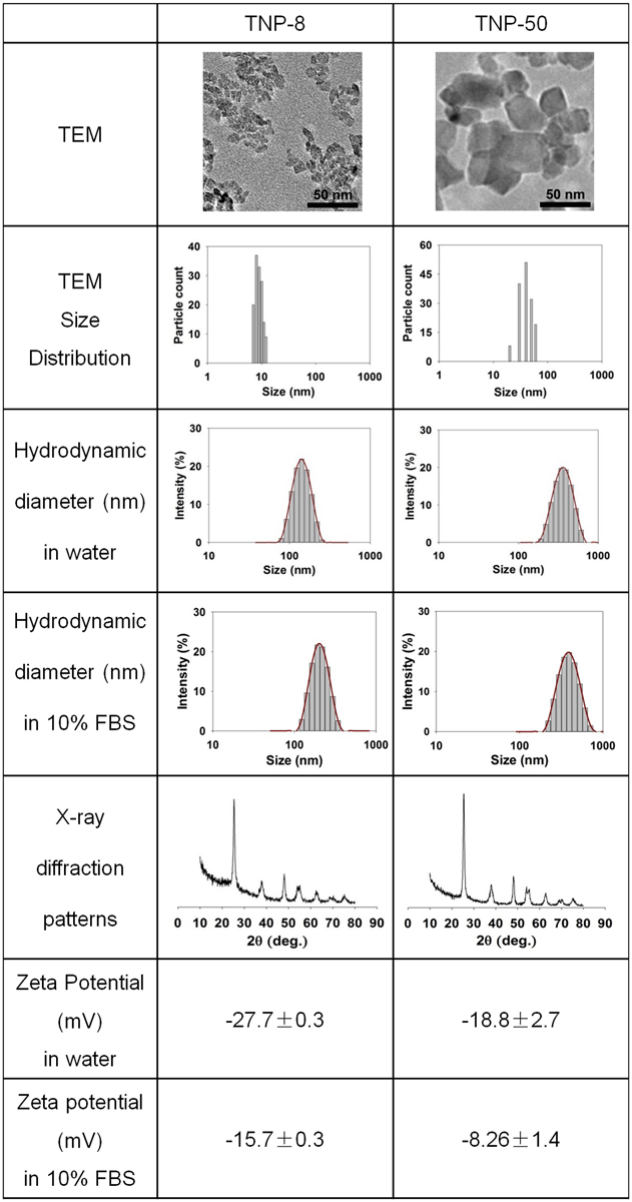
Characterization of TNPs.

The physiochemical properties of nanoparticles are vital to their biological activities [26–28]. Particle size, zeta potential, agglomeration, and crystal structure of TNP-8 and TNP-50 were determined and summarized in Fig 1. Both larger and smaller TNPs had anatase structure as shown by their X-ray diffraction patterns (Fig 1). The TNPs also had similar zeta potential values in water and plasma, suggesting that their surface electrostatic and electrodynamic properties were very similar. In water, nanoparticles of both sizes exhibited a slight aggregation due to the tendency of nanoparticles to minimize their surface energy in aqueous suspension.

**Fig 2:**
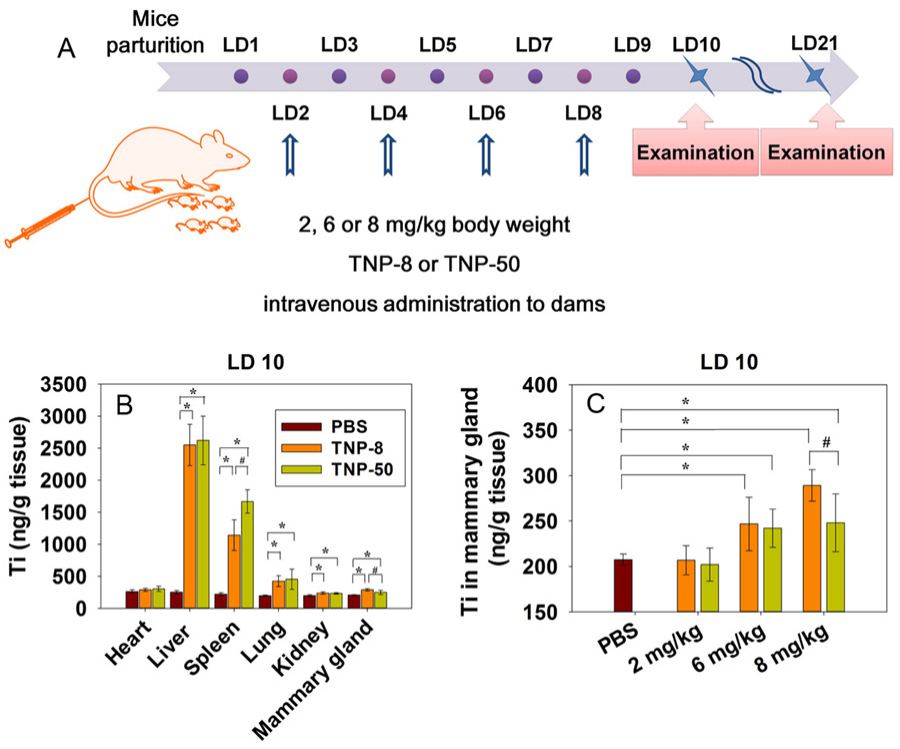
TNP exposure scheme and biodistribution of TNPs in lactating dams after intravenous injection. (A) TNP exposure scheme. Lactating dams received exposure of TNP**-**8 or TNP-50 at lactation day (LD) 2, 4, 6, and 8 at a dose of 2, 6 or 8 mg/kg body weight via intravenous administration. (B) Ti content in major organs of lactating dams at LD 10 after four doses of TNPs (8 mg/kg) at LD 2, 4, 6 and 8. Ti content in PBS group showed the basal Ti level in various organs. (C) Ti content in mammary glands at LD 10 after dams were exposed to four doses of TNPs at indicated doses at LD 2, 4, 6 and 8. The symbol * represents significant difference from the PBS group (P<0.05) and # represents significant difference from the TNP-8 group (P<0.05). In both B and C, data are mean±s.d. (n = 7 per group).

Although medicinal application of TNPs often involves a direct i.v. injection [6], environmental exposures through oral, dermal, or inhalation routes all lead to the absorption of nanoparticles into the circulation followed by the translocation of nanoparticles to various organs [17, 25, 29, 30]. During the lactation period, there is an increased blood flow to the mammary glands. This may facilitate the transfer of nanoparticles from blood to mammary glands [31]. Based on this, we chose i.v. administration for the toxic study of lactating mammary gland. To simulate these processes, we give mice four i.v. injections of TNPs at doses of 2, 6, and 8 mg/kg (Fig 2A).

We first examined the organ accumulation of the TNPs using inductively coupled plasma mass spectrometry (ICP-MS) [25, 32]. TNPs were accumulated more readily in reticuloendothelial (RES) organs, liver and spleen at lactation day 10 (LD 10) as found in other studies [17, 33]. In comparison, the accumulation in the lungs, heart, and kidneys were much less (Fig 2B). This showed that TNP**-**8 and TNP-50 were primarily cleared from blood by RES system organs. The relatively low accumulation of TNPs in the kidney suggested that urinary excretion is not the main excretion pathway for TNPs.

TNPs of both sizes were found accumulated in mammary glands (Fig 2C). Titanium accumulation in mammary gland was more evident for higher exposure groups and for smaller nanoparticles showing that nanoparticles accumulation in mammary gland of lactating mice was size- and dose-dependent (Fig 2C). The internal exposure doses of TNP-8 and -50 in mammary glands at an applied dose of 8 mg/kg were approximately 82 and 35 ng Ti/g tissue, respectively, at LD 10. In comparison, the internal doses of TNP-8 and -50 in liver were 2,299.6 and 2,369.2 ng Ti/g.

The next question was whether the four i.v. injections of TNPs at 8 mg/kg could induce acute systemic toxicity in dams. During the entire lactation period, none of the dams exhibited abnormal behaviors in routine activities. There were no significant changes in body weight (S1A Fig), relative organ weights (S1B and S1C Fig), or food consumption (S1D Fig) compared with the control mice. Pathological examinations of major organs found no TNP-induced tissue injury after exposure at the highest dose (S2 Fig).We also analyzed possible TNP-induced physiological alterations as reflected in blood. At LD 10, there was no abnormal alteration in hematology indicators (S1 Table). The above results indicated that, at 8 mg/kg, the systemic toxicity of these two TNPs to dams was minimal during the lactation period.

Several *in vivo* toxicity studies were reported previously at considerably higher doses and via other administration routes. Intragastric administration of TNPs with a diameter of 5 nm at a dose of 125 or 250 mg/kg to mice induced relative organ weight increases for the liver, kidney, and spleen as well as alterations in hematological characteristics [34]. The intraperitoneal (i.p.) administration of TNPs with a diameter of 100 nm at doses of 1,940 and 2,590 mg/kg to mice caused acute toxicity, as indicated by passive behavior, loss of appetite, tremor and death [32]. The much lower doses used in our study compared to previous studies might account for the lack of systemic toxicity. However, considering the vulnerability of mammary glands and their crucial function in feeding the newborns, it was highly desirable to examine the effects of TNPs on this organ and on pups at a dose that caused no systemic toxicity to dams.

In this study, TNPs caused cell shedding and tight junction disruption in a size-dependent manner. The exposure dose was expressed as particle mass concentration. In nanotoxicity/ nanomedicine studies, dose can also be expressed as particle surface area or particle number [35, 36]. Previous study shows that toxicity is dependent on dosage. Smaller nanoparticle often shows a greater toxicity when using mass concentration in agreement with our findings.

### TNP-induced oxidative stress in mammary glands impairs tight junction of blood-milk barrier

**Fig 3:**
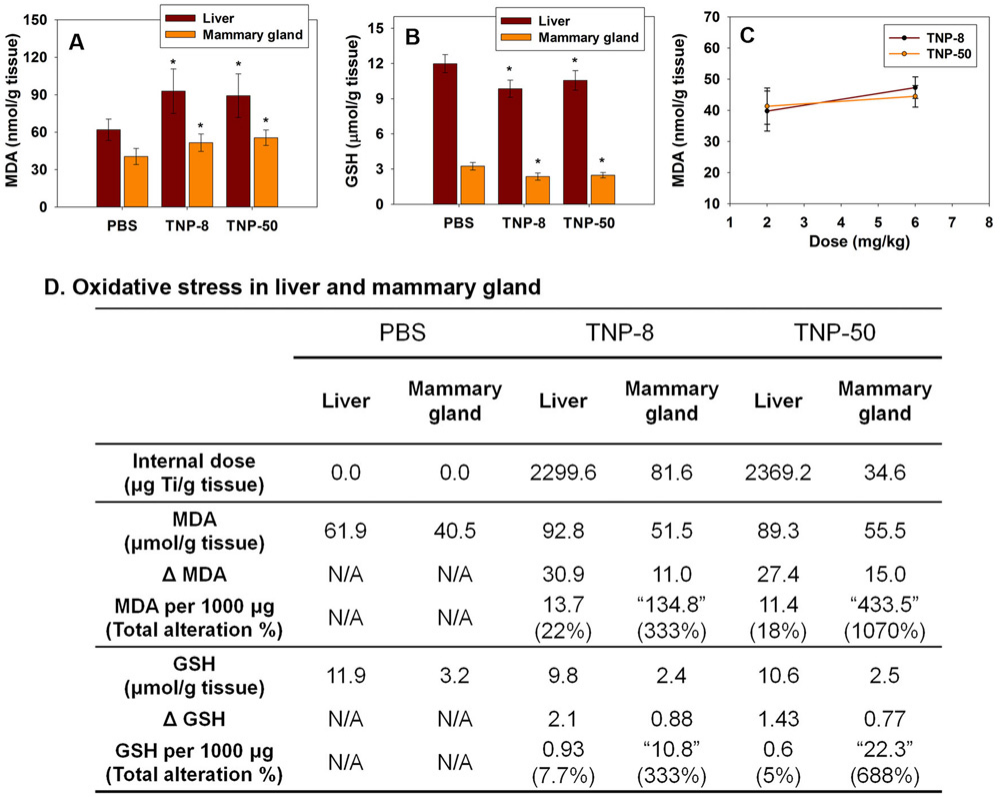
Determination of levels of MDA (A), GSH (B), Linear correlation between dose and MDA in mammary gland (C) and comparison of TNP generated oxidative stress in mammary glands and liver (D) at LD 10 after four doses of TNP exposures (8 mg/kg) at LDs 2, 4, 6 and 8. Seven mice in each group were examined. Data are mean±s.d. (n = 7 per group). Values in "" are hypothetical values when the internal dose in mammary gland were 1000 μg Ti/g tissue. The symbol * represents significant difference from the PBS group (P<0.05). ΔMDA = (Liver MDA in TNP groups)–(Liver MDA in PBS groups). $MDA\;per\;1000\;\mu g\;=\;\frac{\Delta \;MDA}{internal\;dose}\times 1000$. ΔGSH and GSH per 1000 μg were obtained similarly.

The generation of oxidative stress by nanomaterials is suggested to be one of the main reasons for nanoparticle-induced tissue damages in testis [19], brain [27, 37], liver [38, 39], placenta and fetuses [18, 40]. Moreover, neutrophil-induced oxidative stress was found to damage mammary gland tissues in animals with mastitis (inflammation of the breast) [41, 42]. The accumulation of TNPs in lactating mammary glands suggested that TNPs might generate local oxidative stress. To evaluate the oxidative stress level in mammary glands, we analyzed the concentrations of two oxidative stress markers, malondialdehyde (MDA) and glutathione (GSH), in mammary glands. The exposure of the dams to TNP-8 and TNP-50 at 8 mg/kg increased the MDA level and decreased the GSH level in mammary glands (Fig 3A and 3B). The mammary glands exhibited a considerably lower antioxidative capability than the liver, as indicated by a comparison of the net increases in MDA and decreases in GSH in mammary glands and liver when the internal doses of TNPs were normalized to the same value (Fig 3C). As shown in Fig 3C, the TNP-induced oxidative stress in mammary glands would be 15–130 times higher than that in the liver if the internal dose were normalized to the same level. This indicated that mammary glands were more vulnerable to nanoparticle-induced oxidative stress. The lower antioxidative capability of mammary glands, combined with the TNP-induced oxidative stress suggested the possibility of tissue injury in the mammary glands of lactating dams.

**Fig 4:**
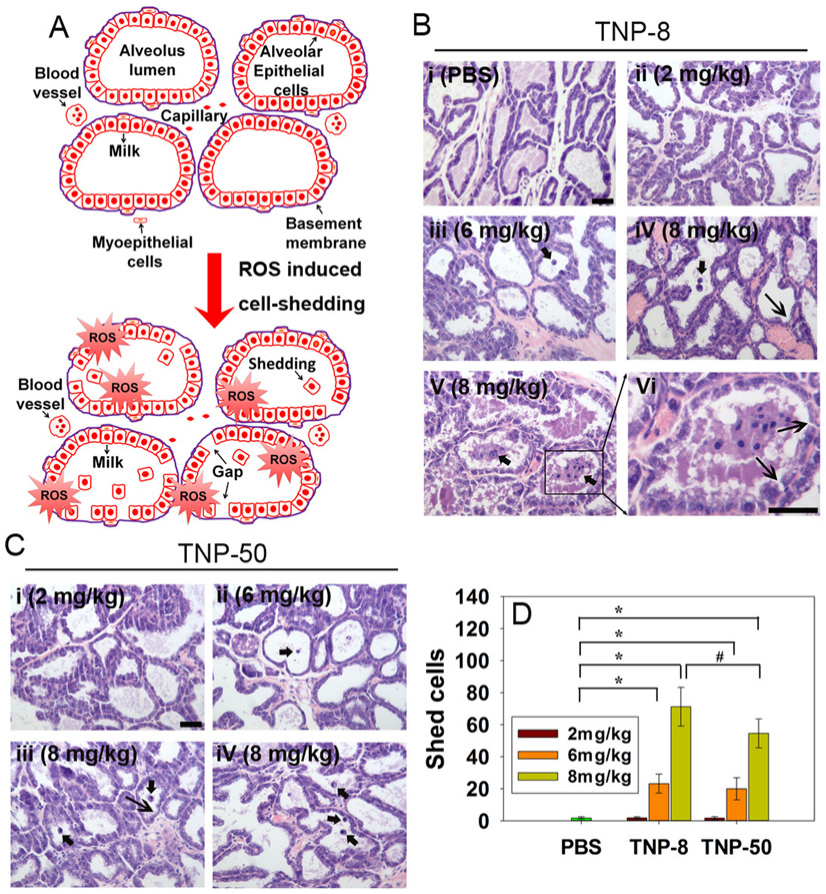
Shedding of mammary alveolar epithelial cells induced by TNPs. A. Schematic anatomical diagrams of lactating mammary glands during lactation period and cartoon indicates that ROS induced cell shedding. B. Representative histological micrographs of the mammary glands 10 days after treatment with (i) PBS; (ii) TNP**-**8 at a dose of 2 mg/kg; (iii) TNP**-**8 at a dose of 6 mg/kg; (iv) TNP**-**8 at a dose of 8 mg/kg. Cell shedding and barrier loosening are noted; (v) Severe cell shedding into alveolar lumen is evident at a dose of 8 mg/kg; (vi) An enlarged view of cell shedding. C. Representative histological micrographs of the mammary glands 10 days after treatment with (i) TNP**-**50 at a dose of 2 mg/kg; (ii) TNP**-**50 at a dose of 6 mg/kg; (iii) TNP**-**50 at a dose of 8 mg/kg; (iv) Cell shedding into alveolar lumen is observed at a dose of 8 mg/kg. D. Quantification of numbers of mammary alveolar epithelial cells shed into the alveolar lumen. There were 7 mice in each experimental group and around 100 alveoli images were examined for each mouse. Scale bar: 40 μm; Narrow arrows indicate gap; Thick arrows indicate cell shedding.

Mammary alveolar epithelial cells are polarized secretory cells (Fig 4A). These cells synthesize milk proteins and secrete milk. There are tight junctions between the basal (blood) and apical (milk) sides of alveolar epithelial cells. The vital function of this barrier is to prohibit the direct paracellular exchange of substances between vascular and milk compartments during the lactation period. Because peak lactation in rodents occurs on LD 10, we first preformed histological examination of the integrity of mammary glands at LD 10 (Fig 4B–4D). The mammary glands of the lactating dams after PBS treatment contained intact alveolar epithelial cells. There was no noticeable cell shedding into the alveolar lumen (Fig 4Bi). After treatment with TNP**-**8 or TNP-50 at 2 mg/kg, the mammary glands showed no pathological alterations compared to the PBS group (Fig 4Bii for TNP**-**8 and Fig 4Ci for TNP**-**50). As the exposure dose increased, more shedding of epithelial cells into the alveolar lumen was observed (Fig 4Biii for TNP**-**8 and Fig 4Cii for TNP**-**50). Cell shedding became more severe when dams were exposed to a higher dose of TNP (Fig 4Biv and v for TNP**-**8 and Fig 4Ciii and iv for TNP**-**50). At the highest dose used (8 mg/kg), hyperplasia and stress-induced adipocytes were observed only for TNP**-**8 treatment (S3A and S3B Fig). The loss of mammary gland epithelial cells may produce defects or gaps in the tight junction of blood-milk barrier. By examining seven mice per exposure group and approximately 100 histological images for each mouse, we quantitatively determined the number of shed cells after each exposure. Our data showed that the smaller TNPs caused more cell shedding than the larger TNPs and cell shedding induced by both TNPs was dose-dependent (Fig 4D).

The mammary alveolar epithelial cells and tight junctions between these cells form an important part of blood-milk barrier [43, 44]. This tight junction is impermeable during the lactation period to prohibit the direct paracellular exchange of substances between the vascular and milk compartments [43]. Tight junction for biological barrier is, however, susceptible to disruption by stimuli such as oxidative stress [45], microbes, or endotoxins [46]. For example, fine particles have been shown to induce ROS and alter the tight junction integrity with associated loss of tight junction proteins such as ZO-1 [47] or both occludin and ZO-1[48].

**Fig 5:**
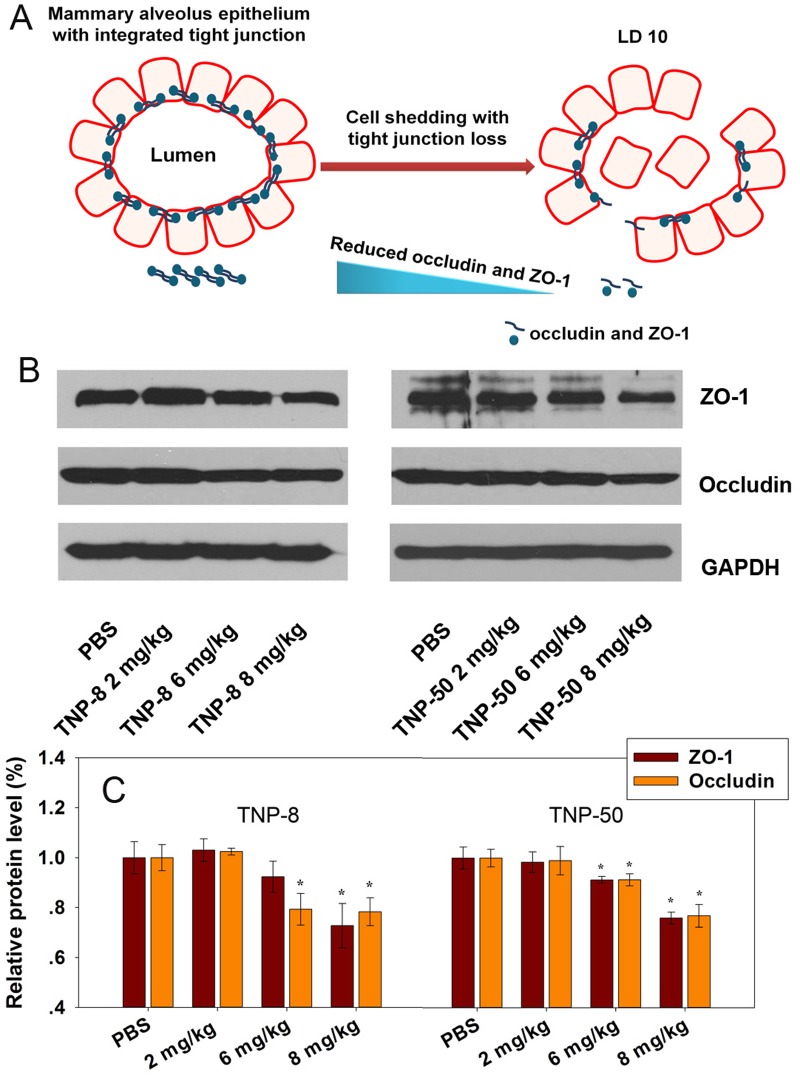
Effects of TNPs on mammary gland tight junction proteins ZO-1 and occludin after four doses of TNPs exposure (2, 6, 8 mg/kg) to dams at LDs 2, 4, 6 and 8. (A) A plausible mechanistic schematic showing TNPs made cell shedding and loose the tight junction between the mammary gland epithelial cells through oxidative stress. (B) Western blot of ZO-1 and occludin in mammary glands. (C) Bar graphs show relative levels of tight junction proteins ZO-1 and occludin by normalizing PBS group to 1.0. The symbol * represents significant difference from the PBS group (P<0.05), (n = 3).

Previous studies have shown that transcytosis of nanoparticles through endothelial cell layers is a rare event [49, 50]. The tight junction proteins ZO-1 and occludin are two key proteins that maintain the integrity of epithelial cell tight junctions in mammary glands [51]. Because TNPs induced local oxidative stress in mammary glands, we hypothesized that this might compromise the tight junctions of the blood-milk barrier by down-regulating the expression of integral proteins occludin and ZO-1 (Fig 5A). To test this hypothesis, we quantitatively analyzed the expression of occludin and ZO-1 in mammary gland tissues using Western blot and band intensity analysis with ImageJ software (Wayne Rasband, NIH, Bethesda, Maryland, USA). We observed a dose-dependent decrease of both proteins (Fig 5B and 5C). These results indicated that exposure of the dams to TNPs caused a reduced expression of key tight junction proteins ZO-1 and occludin and the shedding of epithelial cells in the mammary glands. We speculate that TNP-induced oxidative stress in mammary glands may be associated with cell shedding and the loss of tight junction proteins. However, whether there is casual link between cell shedding and tight junction disruption awaits further investigations. These perturbations significantly compromised the integrity of the blood-milk barrier. As a result, a possibility may exist that TNPs may leak into milk and reach the GI tracts of pups during milk feeding.

### Damaged blood-milk barrier allowed TNPs to enter pup’s GI tracts possibly through milk feeding

**Fig 6:**
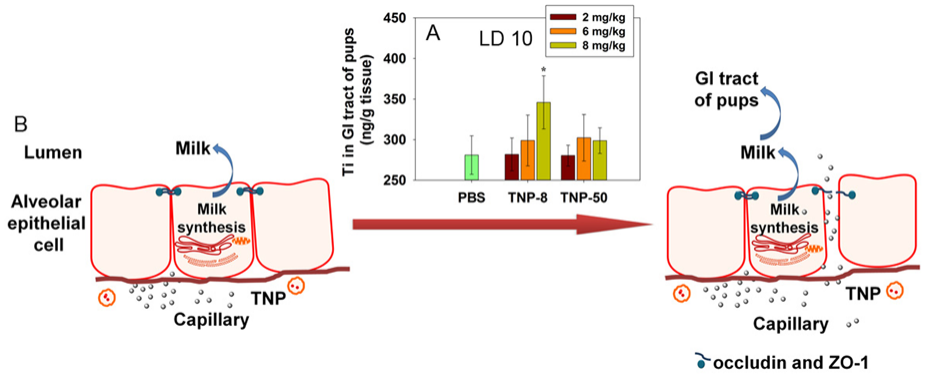
The Ti content in pups’ GI tract and a cartoon presentation showing that TNPs cross the damaged tight junction entering the milk in lactating dams. (A) Ti content in pups’ GI tract at LD 10 after four doses of TNP exposures to lactating dams at LD 2, 4, 6 and 8. Symbol * represents significant difference from the PBS group (P<0.05). GI tract tissues from eight pups were collected per dam and pooled. There are five dams in each group. Data are mean±s.d. (B) A cartoon presentation showing that TNPs cross the damaged tight junction entering the milk in lactating dams.

Milk production and secretion are key functions of mammary glands. The disruption of tight junctions in mammary glands caused the transfer of small molecules from blood and interstitial spaces to milk [52]. It is intuitive that damage to the blood-milk barrier may also cause the transfer of TNPs from blood to milk. To demonstrate this transfer, it would be highly desirable to show the existence of TNPs in maternal milk. Technically, this experiment is very challenging because the tiny amount of secreted milk from a mouse is difficult to collect for ICP-MS analysis. Because milk is the only food source for newborn pups, TNPs may enter the pup’s GI tract through the milk feeding. To test this possibility, we analyzed the Ti content in pups’ GI tracts at LD 10 using ICP-MS (five dams per group, combined tissues from eight pups per dam). Our results demonstrated that Ti was accumulated in the GI tracts of the pups at LD 10 when TNP-8 particles were administered to the dams at a dose of 8 mg/kg (Fig 6A). In contrast, accumulation of Ti was not evident for larger TNP**-**50 or lower doses of TNP**-**8 particles. By analyzing Ti content in pups, we found that Ti content in GI tracts of pups is generally higher with a small standard deviation. Since the main activity of pups in lactation period was milk sucking, the probability of Ti transmission through random contamination from dam’s feces and urine was very low. The observed TNP**-**8 transfer from dams to pups during milk feeding at a maternal exposure dose of 8 mg/kg was consistent with the observed higher oxidative stress induction (Fig 3C) and tight junction damage (Figs 4B and 5B) by TNP**-**8 at this dose (Fig 6B). Nanoparticles have also been found to damage other biological barriers, such as the placental barrier [18, 53] and blood-testis barrier [20].

### Milk nutrient and pup development not compromised during lactation period

Dam’s milk is the only food source for pups during lactation period. Nutrient proteins in the milk play at least three crucial roles: providing nutrition, defending pups against infection, and facilitating the development of physiological functions [54]. For example, β-casein plays critical roles in calcium solubility and absorption. The expression of the β-casein gene has been considered an indicator of milk protein synthesis [55]. α-Lactalbumin is a common whey protein that plays multiple protective functions in the newborns [56]. Lactoferrin is an antimicrobial component [57, 58]. Epithelial growth factor (EGF) functions in stimulating the growth of the gut and various epidermal/epithelial tissues as well as preventing bacterial infection [59].

The detection of TNPs in pup’s GI tract suggested that TNPs might enter milk and affect milk components and nutrition. Therefore, we next investigated the impact of TNPs on nutrient quality of milk by harvesting milk from the mammary gland tissue of lactating dams at LD 10 and analyzing key nutrient proteins in the milk.

**Fig 7:**
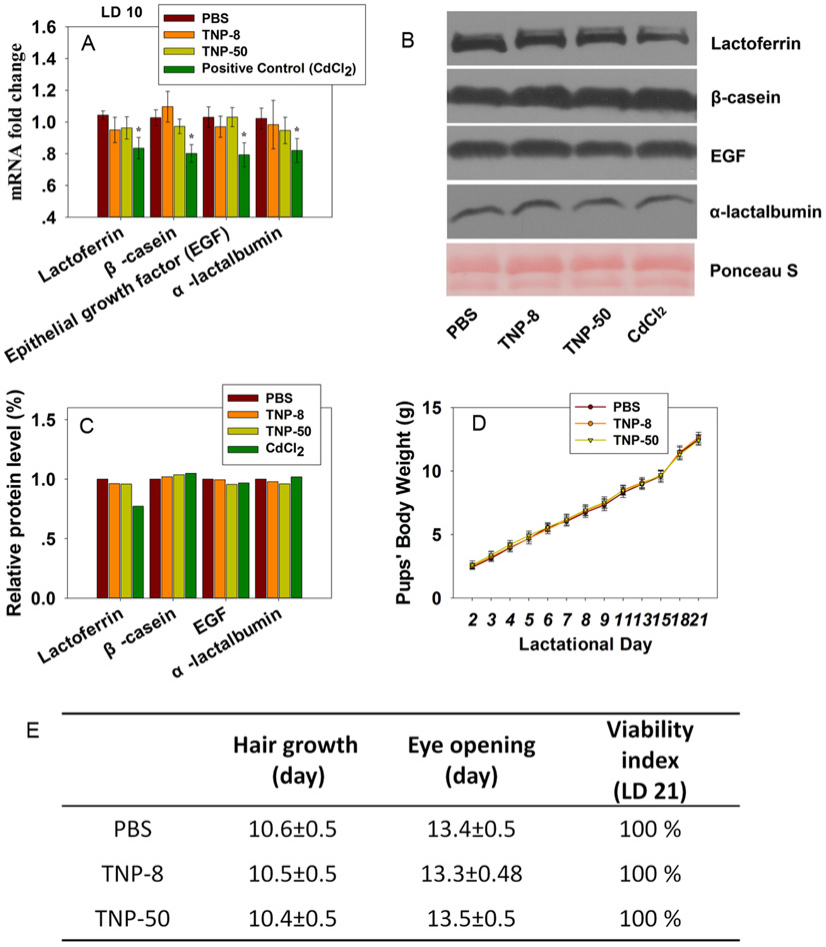
Analysis of nutrient quality of milk in the lactating dams (A, B and C) and effects of TNP exposures to dams on developmental landmarks of pups during lactation period (D, E). (A) The mRNA levels of β-casein, lactoferrin, α-lactalbumin and epidermal growth factor in mammary gland tissue were analyzed at LD 10 by quantitative PCR assay after dosing of TNPs at 8 mg/kg to dams. Data are mean±sd. (n = 4) (B) Western blot of key milk proteins. (C) Bar graphs show relative levels of β-casein, lactoferrin, α-lactalbumin and epidermal growth factor by normalizing PBS group to 1.0. (D) Body weight gains of pups during the lactating period when dams were dosed with PBS, TNP-8 or TNP-50 (8 mg/kg BW) at LDs 2, 4, 6 and 8. (n = 7) (E) Effects of TNP treatments on key developmental landmarks of pups during lactation period. The symbol * represents significant difference from the PBS group (P<0.05).

Although, the positive control, CdCl_2_ (2 mg/kg), downregulated the transcription of the genes encoding β-casein, α-lactalbumin, lactoferrin, and EGF from quantitative PCR analysis (Fig 7A) as well as the expression of lactoferrin protein (Fig 7B and 7C), TNPs did not reduce the expression of key milk components at both mRNA and protein levels (Fig 7A–7C) when dams were exposed to TNPs at a dose of 8 mg/kg. These data indicated that the leakage of TNPs into milk did not deteriorate the nutrient quality of the milk. Milk supplies all of the nutrients required for neonatal growth [12], cognitive and psychosocial development [14], and immune defense [13]. Approximately 80–95% of the proteins in milk are synthesized in mammary alveolar epithelial cells [60]. The absence of a short-term negative effect of TNPs on the nutrient quality of milk was observed in this study. This result indicated that the TNPs had minimal effects on the synthesis of milk proteins even though they disrupted the blood-milk barrier.

However, the development of pups during the lactation period might still be a concern for at least three reasons: (1) possible alterations in other protein components in milk; (2) the quantity of secreted milk might be reduced by TNPs; and (3) the TNP leakage to the GI tracts of pups might cause damages to pup’s systems. Therefore, we next examined the developmental landmarks of pups during the lactation period.

To accomplish this, we recorded pups’ body weight gains (Fig 7D), viability at weaning (LD 21), and other major physical developmental landmarks (Fig 7E). All the pups gained weight at the same rate as the pups in the control group. The average days for eye opening and hair growth for pups from the TNP-treated dams were nearly identical to those of the control pups. All groups also had identical weaning day viability (100%).

Regarding the effects of TNP accumulation in the mammary glands of dams on their ability to secrete milk, we did not have a quantitative method for measuring milk secretion. However, based on the comparable body weight gains in the pups from the TNP-treated and control dams, we inferred that the quantity of secreted milk was not likely reduced by TNP accumulation in the mammary glands of dams during the lactation period.

Despite the lack of effects to pups during lactation period, the long term effects of these exposures cannot be ignored. Carbon nanoparticles administered to pregnant mice have been reported to cause defects in the reproductive systems of male offspring [61]. It was also reported that a decline of spatial recognition memory and learning of offspring caused by exposure of TiO_2_ nanoparticles to dams during lactation period [62]. This is probably due to the fact that hippocampal development and neurobehavioral development occur during lactation period rather than prenatal period [16].

## Conclusion

In summary, we use lactating mice as a model in this investigation to examine the size- and dose-dependent effects of TNPs on lactating dams and pups. After i.v. administration, TNPs accumulated in the mammary glands, in addition to other organs, in a size-dependent manner. Nanoparticles generate oxidative stress in mammary glands and cause damages to the tight junction in blood-milk barrier as indicated by the shedding of epithelial cells. TNP-induced mammary gland damage and the leakage of nanoparticles to pups through milk feeding are highly dependent on the size of the nanoparticles. The accumulation of TNPs in the GI tracts of pups was observed only for smaller TNP-8 particles after milk feeding. Although the primary sizes of TNPs are discussed, we emphasize here that nanoparticles exist mainly as aggregates and they are actually more biologically relevant. Under our exposure scheme and doses, the nutrient proteins in milk were not compromised and the developmental landmarks of the pups were not affected through the weaning day. However, the effects of higher doses and effects of other nanoparticles still need to be elucidated and the long-term effects of such exposures on offsprings remain to be clarified.

## Supporting Information

S1 Fig Effects of TNP exposure on dams during lactation. (A) Change in body weight of dams during and after exposures to four doses of TNPs (8 mg/kg) at LD 2, 4, 6, and 8. Relative organ weights of dams at LD 10 (B) and 21 (C) after exposures to four doses of TNPs (8 mg/kg) at LD 2, 4, 6 and 8. (D) Food consumption by dams during whole lactation period (21 days) after exposures to four doses of TNPs (8 mg/kg) at LD 2, 4, 6 and 8. Seven mice in each group were examined. Data are mean±s.d. (n = 7 per group).
(TIF)

S2 Fig Histopathological micrographs of major organs in dams at LD 10 after exposures to four doses of TNP (8 mg/kg). Seven dams in each group were used for histological examination.
(TIF)

S3 Fig Pathology of mammary glands of TNP-8 at a dose of 8 mg/kg. Red triangles and arrows indicate stress-induced adipocytes and hyperplasia respectively.
(TIF)

S1 File Additional materials and methods. (DOCX)

S1 Table Blood biochemistry (A) and hematology (B) of dams after TNP-8 and -50 exposure (8 mg/kg) at LD 10. (DOCX)

## References

[1] Project on Emerging Nanotechnologies (2014). Consumer Products Inventory. Available: http://www.nanotechproject.org/cpi/. Accessed 8 September 2014.

[2] Zhu HY, Gao XP, Lan Y, Song DY, Xi YX, Zhao JC. Hydrogen titanate nanofibers covered with anatase nanocrystals: A delicate structure achieved by the wet chemistry reaction of the titanate nanofibers. J Am Chem Soc. 2004;126(27):8380–8381. 10.1021/ja048204t 15237986

[3] Zhu HY, Lan Y, Gao XP, Ringer SP, Zheng ZF, Song DY, et al Phase transition between nanostructures of titanate and titanium dioxides via simple wet-chemical reactions. J Am Chem Soc. 2005;127(18):6730–6736. 10.1021/ja044689+ 15869295

[4] Schilling K, Bradford B, Castelli D, Dufour E, Nash JF, Pape W, et al Human safety review of "nano" titanium dioxide and zinc oxide. Photochem Photobiol Sci. 2010;9(4):495–509. 10.1039/b9pp00180h 20354643

[5] Chen XX, Cheng B, Yang YX, Cao A, Liu JH, Du LJ, et al Characterization and Preliminary Toxicity Assay of Nano-Titanium Dioxide Additive in Sugar-Coated Chewing Gum. Small. 2013;9(9–10):1765–1774. 10.1002/smll.201201506 23065899

[6] Seo JW, Chung H, Kim MY, Lee J, Choi IH, Cheon J. Development of Water-Soluble Single-Crystalline TiO2 Nanoparticles for Photocatalytic Cancer-Cell Treatment. Small. 2007;3(5):850–853. 10.1002/smll.200600488 17385208

[7] Yu JC, Ho WK, Yu JG, Yip H, Wong PK, Zhao JC. Efficient visible-light-induced photocatalytic disinfection on sulfur-doped nanocrystalline titania. Environ Sci Technol. 2005;39(4):1175–1179. 10.1021/es035374h 15773492

[8] Jacoby WA, Maness PC, Wolfrum EJ, Blake DM, Fennell JA. Mineralization of bacterial cell mass on a photocatalytic surface in air. Environ Sci Technol. 1998;32(17):2650–2653. 10.1021/es980036f

[9] Chen H, Nanayakkara CE, Grassian VH. Titanium Dioxide Photocatalysis in Atmospheric Chemistry. Chem Rev. 2012;112(11):5919–5948. 10.1021/cr3002092 23088691

[10] Kaegi R, Ulrich A, Sinnet B, Vonbank R, Wichser A, Zuleeg S, et al Synthetic TiO(2) nanoparticle emission from exterior facades into the aquatic environment. Environ Pollut. 2008;156(2):233–239. 10.1016/j.envpol.2008.08.004 18824285

[11] Lovern SB, Strickler JR, Klaper R. Behavioral and physiological changes in Daphnia magna when exposed to nanoparticle suspensions (titanium dioxide, nano-C-60, and C(60)HxC(70)Hx). Environ Sci Technol. 2007;41(12):4465–4470. 10.1021/es062146p 17626453 PMC2556055

[12] Dewey KG. Cross-cultural patterns of growth and nutritional status of breastfed infants. Am J Clin Nutr. 1998;67(1):10–17. 9440369 10.1093/ajcn/67.1.10

[13] Labbok MH, Clark D, Goldman AS. Science and society—Breastfeeding: maintaining an irreplaceable immunological resource. Nat Rev Immunol. 2004;4(7):565–572. 10.1038/nri1393 15229475

[14] Anderson JW, Johnstone BM, Remley DT. Breast-feeding and cognitive development: a meta-analysis. Am J Clin Nutr. 1999;70(4):525–535 10500022 10.1093/ajcn/70.4.525

[15] Sumner SCJ, Fennell TR, Snyder RW, Taylor GF, Lewin AH. Distribution of carbon-14 labeled C60 (C-14 C60) in the pregnant and in the lactating dam and the effect of C60 exposure on the biochemical profile of urine. J Appl Toxicol. 2010;30(4):354–360. 10.1002/jat.1503 20063269

[16] Gao X, Yin S, Tang M, Chen J, Yang Z, Zhang W, et al Effects of Developmental Exposure to TiO2 Nanoparticles on Synaptic Plasticity in Hippocampal Dentate Gyrus Area: an In Vivo Study in Anesthetized Rats. Biol Trace Elem Res. 2011;143(3):1616–1628. 10.1007/s12011-011-8990-4 21331565

[17] Zhang C, Zhang Q, Zhang Y, Yan B. Toward a Better Understanding of Pharmacokinetics of Nanomaterials. Curr Pharm Des. 2013;19(37):6667–6680. 10.2174/1381612811319370010 23621528

[18] Yamashita K, Yoshioka Y, Higashisaka K, Mimura K, Morishita Y, Nozaki M, et al Silica and titanium dioxide nanoparticles cause pregnancy complications in mice. Nat Nanotechnol. 2011;6(5):321–328. 10.1038/nnano.2011.41 21460826

[19] Bai YH, Zhang Y, Zhang JP, Mu QX, Zhang WD, Butch ER, et al Repeated administrations of carbon nanotubes in male mice cause reversible testis damage without affecting fertility. Nat Nanotechnol. 2010;5(9):683–689. 10.1038/nnano.2010.153 20693989 PMC2934866

[20] Li WQ, Wang F, Liu ZM, Wang YC, Wang J, Sun F. Gold Nanoparticles Elevate Plasma Testosterone Levels in Male Mice without Affecting Fertility. Small. 2013;9(9–10):1708–1714. 10.1002/smll.201201079 22911975

[21] Su G, Zhou H, Mu Q, Zhang Y, Li L, Jiao P, et al Effective Surface Charge Density Determines the Electrostatic Attraction between Nanoparticles and Cells. J Phys Chem C. 2012;116(8):4993–4998. 10.1021/jp211041m

[22] DePeters EJ, Hovey RC. Methods for Collecting Milk from Mice. J Mammary Gland Biol Neoplasia. 2009;14(4):397–400. 10.1007/s10911-009-9158-0 19936987 PMC2789213

[23] Deng X, Wu F, Liu Z, Luo M, Li L, Ni Q, et al The splenic toxicity of water soluble multi-walled carbon nanotubes in mice. Carbon. 2009;47(6):1421–1428. 10.1016/j.carbon.2008.12.032 19059422

[24] Hougaard K, Jackson P, Jensen K, Sloth J, Loschner K, Larsen E, et al Effects of prenatal exposure to surface-coated nanosized titanium dioxide (UV-Titan). A study in mice. Part Fibre Toxicol. 2010;7(1):16 10.1186/1743-8977-7-16 20546558 PMC2908059

[25] Wang J, Zhou G, Chen C, Yu H, Wang T, Ma Y, et al Acute toxicity and biodistribution of different sized titanium dioxide particles in mice after oral administration. Toxicol Lett. 2007;168(2):176–185. 10.1016/j.toxlet.2006.12.001 17197136

[26] Yeo MK, Kang M. The biological toxicities of two crystalline phases and differential sizes of TiO2 nanoparticles during zebrafish embryogenesis development. Mol Cell Toxicol. 2012;8(4):317–326. 10.1007/s13273-012-0039-z

[27] Wang J, Liu Y, Jiao F, Lao F, Li W, Gu Y, et al Time-dependent translocation and potential impairment on central nervous system by intranasally instilled TiO2 nanoparticles. Toxicology. 2008;254(1–2):82–90. 10.1016/j.tox.2008.09.014 18929619

[28] Zhang L, Bai R, Li B, Ge C, Du J, Liu Y, et al Rutile TiO2 particles exert size and surface coating dependent retention and lesions on the murine brain. Toxicol Lett. 2011;207(1):73–81. 10.1016/j.toxlet.2011.08.001 21855616

[29] Wu J, Liu W, Xue C, Zhou S, Lan F, Bi L, et al Toxicity and penetration of TiO2 nanoparticles in hairless mice and porcine skin after subchronic dermal exposure. Toxicol Lett. 2009;191(1):1–8. 10.1016/j.toxlet.2009.05.020 19501137

[30] Cho WS, Kang BC, Lee JK, Jeong J, Che JH, Seok SH. Comparative absorption, distribution, and excretion of titanium dioxide and zinc oxide nanoparticles after repeated oral administration. Part Fibre Toxicol. 2013;10(1):9 10.1186/1743-8977-10-9 23531334 PMC3616827

[31] Williamson DH, Lund P, Evans RD. Substrate selection and oxygen uptake by the lactating mammary gland. Proc Nutr Soc. 1995;54(01):165–175. 10.1079/PNS19950046 7568251

[32] Chen J, Dong X, Zhao J, Tang G. In vivo acute toxicity of titanium dioxide nanoparticles to mice after intraperitioneal injection. J Appl Toxicol. 2009;29(4):330–337. 10.1002/jat.1414 19156710

[33] Xie G, Wang C, Sun J, Zhong G. Tissue distribution and excretion of intravenously administered titanium dioxide nanoparticles. Toxicol Lett. 2011;205(1):55–61. 10.1016/j.toxlet.2011.04.034 21600967

[34] Duan Y, Liu J, Ma L, Li N, Liu H, Wang J, et al Toxicological characteristics of nanoparticulate anatase titanium dioxide in mice. Biomaterials. 2010;31(5):894–899. 10.1016/j.biomaterials.2009.10.003 19857890

[35] Perrault SD, Walkey C, Jennings T, Fischer HC, Chan WCW. Mediating Tumor Targeting Efficiency of Nanoparticles Through Design. Nano Lett. 2009;9(5):1909–1915. 10.1021/nl900031y 19344179

[36] Gosens I, Mathijssen LEAM, Bokkers BGH, Muijser H, Cassee FR. Comparative hazard identification of nano- and micro-sized cerium oxide particles based on 28-day inhalation studies in rats. Nanotoxicology. 2014;8(6):643–653. 10.3109/17435390.2013.815814 23768316

[37] Ma L, Liu J, Li N, Wang J, Duan Y, Yan J, et al Oxidative stress in the brain of mice caused by translocated nanoparticulate TiO2 delivered to the abdominal cavity. Biomaterials. 2010;31(1):99–105. 10.1016/j.biomaterials.2009.09.028 19783296

[38] Yang ST, Wang X, Jia G, Gu Y, Wang T, Nie H, et al Long-term accumulation and low toxicity of single-walled carbon nanotubes in intravenously exposed mice. Toxicol Lett. 2008;181(3):182–189. 10.1016/j.toxlet.2008.07.020 18760340

[39] Liu H, Ma L, Liu J, Zhao J, Yan J, Hong F. Toxicity of nano-anatase TiO2 to mice: Liver injury, oxidative stress. Toxicol Environ Chem. 2010;92(1):175–186. 10.1080/02772240902732530

[40] Pietroiusti A, Massimiani M, Fenoglio I, Colonna M, Valentini F, Palleschi G, et al Low Doses of Pristine and Oxidized Single-Wall Carbon Nanotubes Affect Mammalian Embryonic Development. ACS Nano. 2011;5(6):4624–4633. 10.1021/nn200372g 21615177

[41] Sordillo LM, Aitken SL. Impact of oxidative stress on the health and immune function of dairy cattle. Vet Immunol Immunopathol. 2009;128(1–3):104–109. 10.1016/j.vetimm.2008.10.305 19027173

[42] Lykkesfeldt J, Svendsen O. Oxidants and antioxidants in disease: Oxidative stress in farm animals. Vet J. 2007;173(3):502–511. 10.1016/j.tvjl.2006.06.005 16914330

[43] McManaman JL, Neville MC. Mammary physiology and milk secretion. Adv Drug Delivery Rev. 2003;55(5):629–641. 10.1016/s0169-409x(03)00033-4 12706546

[44] Burton JL, Erskine RJ. Immunity and mastitis Some new ideas for an old disease. Vet Clin North Am Food Anim Pract. 2003;19(1):1–45. 10.1016/S0749-0720(02)00073-7 12682934

[45] Rao R. Oxidative stress-induced disruption of epithelial and endothelial tight junctions. Front Biosci. 2008;13:7210–7226. 10.2741/3223 18508729 PMC6261932

[46] Bonazzi M, Cossart P. Impenetrable barriers or entry portals? The role of cell-cell adhesion during infection. J Cell Biol. 2011;195(3):349–358. 10.1083/jcb.201106011 22042617 PMC3206337

[47] Wang T, Wang L, Moreno-Vinasco L, Lang GD, Siegler JH, Mathew B, et al Particulate matter air pollution disrupts endothelial cell barrier via calpain-mediated tight junction protein degradation. Part Fibre Toxicol. 2012;9(1):35 10.1186/1743-8977-9-35 22931549 PMC3489700

[48] Mutlu E, Engen P, Soberanes S, Urich D, Forsyth C, Nigdelioglu R, et al Particulate matter air pollution causes oxidant-mediated increase in gut permeability in mice. Part Fibre Toxicol. 2011;8(1):19 10.1186/1743-8977-8-19 21658250 PMC3132719

[49] He B, Lin P, Jia Z, Du W, Qu W, Yuan L, et al The transport mechanisms of polymer nanoparticles in Caco-2 epithelial cells. Biomaterials. 2013;34(25):6082–6098. 10.1016/j.biomaterials.2013.04.053 23694903

[50] Ye D, Raghnaill MN, Bramini M, Mahon E, Aberg C, Salvati A, et al Nanoparticle accumulation and transcytosis in brain endothelial cell layers. Nanoscale. 2013;5(22):11153–11165. 10.1039/C3NR02905K 24077327

[51] Stull MA, Pai V, Vomachka AJ, Marshall AM, Jacob GA, Horseman ND. Mammary gland homeostasis employs serotonergic regulation of epithelial tight junctions. Proc Natl Acad Sci U S A. 2007;104(42):16708–16713. 10.1073/pnas.0708136104 17940054 PMC2034263

[52] Stelwagen K, Davis SR, Farr VC, Prosser CG, Sherlock RA. Mammary Epithelial Cell Tight Junction Integrity and Mammary Blood Flow During an Extended Milking Interval in Goats. J Dairy Sci. 1994;77(2):426–432. 10.3168/jds.S0022-0302(94)76969-1 8182166

[53] Tian X, Zhu M, Du L, Wang J, Fan Z, Liu J, et al Intrauterine Inflammation Increases Materno-Fetal Transfer of Gold Nanoparticles in a Size-Dependent Manner in Murine Pregnancy. Small. 2013;9(14):2432–2439. 10.1002/smll.201300817 23761193

[54] Lonnerdal B. Nutritional and physiologic significance of human milk proteins. Am J Clin Nutr. 2003;77(6):1537S–1543S. 12812151 10.1093/ajcn/77.6.1537S

[55] Wheeler TT, Callaghan MR, Davis SR, Prosser CG, Wilkins RJ. Milk Protein Synthesis, Gene Expression, and Hormonal Responsiveness in Primary Cultures of Mammary Cells from Lactating Sheep. Exp Cell Res. 1995;217(2):346–354. 10.1006/excr.1995.1096 7698235

[56] Lönnerdal B, Lien EL. Nutritional and Physiologic Significance of α-Lactalbumin in Infants. Nutr Rev. 2003;61(9):295–305. 10.1301/nr.2003.sept.295-305 14552064

[57] Ward PP, Paz E, Conneely OM. Multifunctional roles of lactoferrin: a critical overview. Cell Mol Life Sci. 2005;62(22):2540–2548. 10.1007/s00018-005-5369-8 16261256 PMC11139086

[58] Valenti P, Antonini G. Lactoferrin: an important host defence against microbial and viral attack. Cell Mol Life Sci. 2005;62(22):2576–2587. 10.1007/s00018-005-5372-0 16261253 PMC11139069

[59] D'Alessandro A, Scaloni A, Zolla L. Human Milk Proteins: An Interactomics and Updated Functional Overview. J Proteome Res. 2010;9(7):3339–3373. 10.1021/pr100123f 20443637

[60] Burgoyne R, Duncan J. Secretion of Milk Proteins. J Mammary Gland Biol Neoplasia. 1998;3(3):275–286. 10.1023/A:1018763427108 10819514

[61] Yoshida S, Hiyoshi K, Oshio S, Takano H, Takeda K, Ichinose T. Effects of fetal exposure to carbon nanoparticles on reproductive function in male offspring. Fertil Steril. 2010;93(5):1695–1699. 10.1016/j.fertnstert.2009.03.094 19446808

[62] Mohammadipour A, Hosseini M, Fazel A, Haghir H, Rafatpanah H, Pourganji M, et al The effects of exposure to titanium dioxide nanoparticles during lactation period on learning and memory of rat offspring. Toxicol Ind Health. 2013 10.1177/0748233713498440 24081627

