# Supplementary figures and images for: Induction of Size-Dependent Breakdown of Blood-Milk Barrier in Lactating Mice by TiO2 Nanoparticles
Source: PLoS One. 2015 Apr 7;10(4):e0122591. doi: 10.1371/journal.pone.0122591 (PMC4388820; doi:10.1371/journal.pone.0122591)

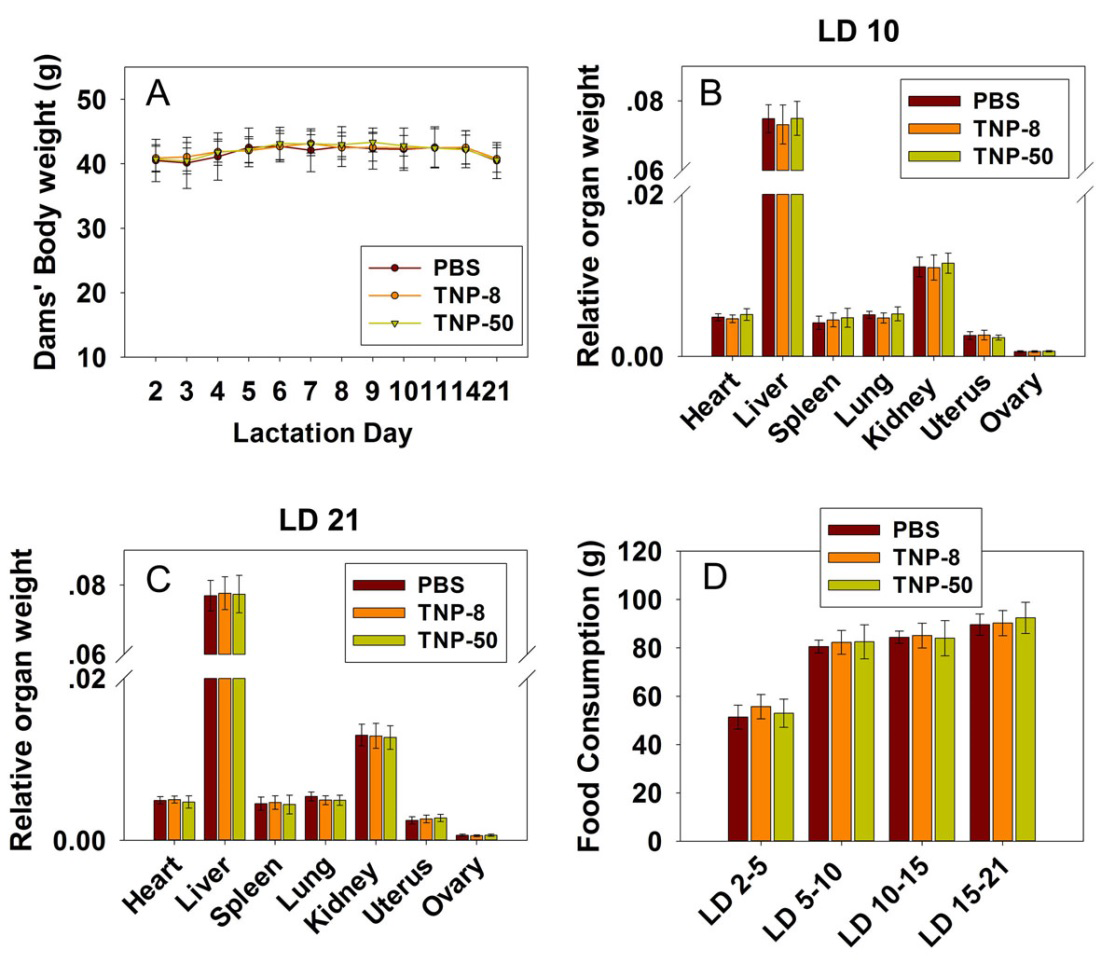

Supplement: S1 Fig — (A) Change in body weight of dams during and after exposures to four doses of TNPs (8 mg/kg) at LD 2, 4, 6, and 8. Relative organ weights of dams at LD 10 (B) and 21 (C) after exposures to four doses of TNPs (8 mg/kg) at LD 2, 4, 6 and 8. (D) Food consumption by dams during whole lactation period (21 days) after exposures to four doses of TNPs (8 mg/kg) at LD 2, 4, 6 and 8. Seven mice in each group were examined. Data are mean±s.d. (n = 7 per group). (TIF) [file pone.0122591.s001.tif]

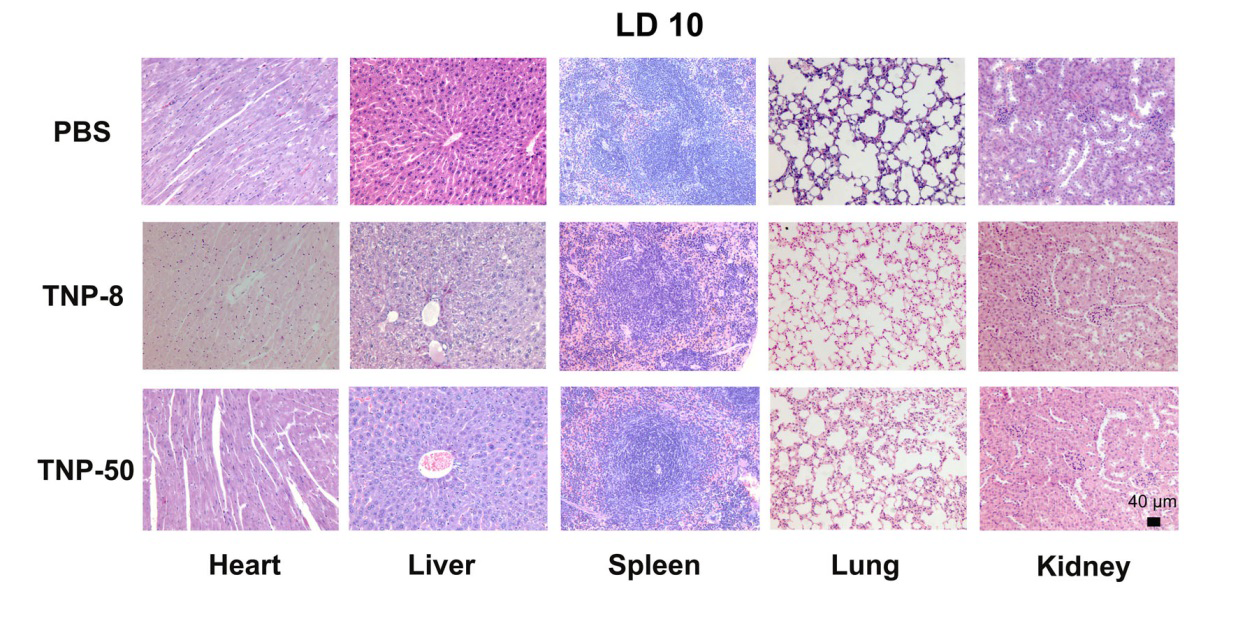

Supplement: S2 Fig — Seven dams in each group were used for histological examination. (TIF) [file pone.0122591.s002.tif]

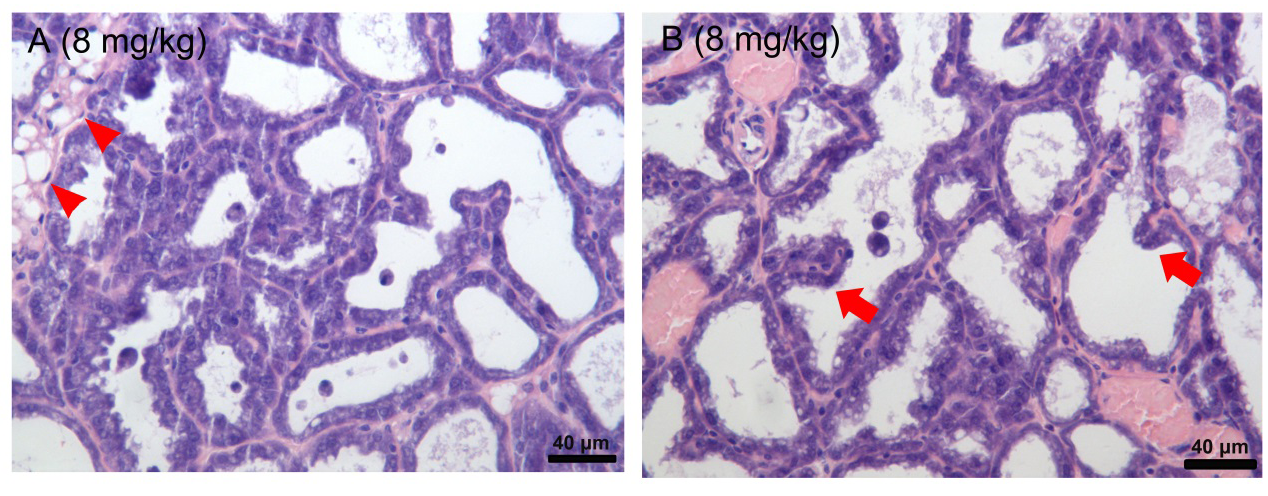

Supplement: S3 Fig — Red triangles and arrows indicate stress-induced adipocytes and hyperplasia respectively. (TIF) [file pone.0122591.s003.tif]
