# Supplementary material for: Induction of Size-Dependent Breakdown of Blood-Milk Barrier in Lactating Mice by TiO2 Nanoparticles
Source: PLoS One. 2015 Apr 7;10(4):e0122591. doi: 10.1371/journal.pone.0122591 (PMC4388820; doi:10.1371/journal.pone.0122591)
Supplement: S1 File — (DOCX) [file pone.0122591.s004.docx]

**Induction of Size-Dependent Breakdown of Blood-Milk Barrier in Lactating Mice by TiO_2_ Nanoparticles**

Chengke Zhang, Shumei Zhai, Ling Wu, Yuhong Bai, Jianbo Jia, Yi Zhang, Bin Zhang, Bing Yan*

**Supporting Information**

**Table of contents**

1. S1 File. Additional materials and methods
2. S1 Fig. Effects of TNP exposure on dams during lactation.
3. S2 Fig. Histopathological micrographs of major organs in dams at LD 10 after exposures to four doses of TNP (8 mg/kg).
4. S1Table. Blood biochemistry and hematology of dams after TNP-8 and -50 exposure.
5. S3 Fig. Pathology of mammary glands of TNP-8 at a dose of 8 mg/kg.
6. **Additional materials and methods**
   1. **Mice care.** Mice were individually housed in individually ventilated cages (32.5×21×18 cm) with sterile wood shavings as bedding. Mice were given a standard pellet diet (Beijing Keao Xieli Feed Co. Ltd., Beijing, China). All mice were maintained in a temperature (22±2℃) and humidity (50±5% relative humidity) controlled room on 12/12 h light-dark cycle and given food and water *ad libitum*.
   2. **TNPs’ characterization.** The morphology of the nanoparticles was analyzed using a JEM-1011 transmission electron microscope (JEOL Ltd., Tokyo, Japan). Prior to TEM observation, the dry powder of TNPs was dispersed in water and diluted to 100 μg/mL and the samples were placed on carbon-coated copper TEM grids (Beijing Xinxing Braim Technology Co.,Ltd., China) drying at room temperature overnight. The nanoparticle size distribution was estimated by analyzing approximately 150 particles using Image-Pro Plus 5.0 software (Media Cybernetics, Silver Spring, MD, USA).

The size distribution in aqueous solution and the surface electrostatic properties of the nanoparticles were characterized by dynamic light scattering and zeta potential measurements using a Zetasizer Nano-ZS (Malvern Instruments, Worcestershire, UK). The data were collected at 25℃ and the average zeta potential and dynamic size values were calculated from three independent measurements.

The crystalline phases were determined by powder X-ray diffractometer (Advance D8, Bruker, Karlsruhe, Germany). XRD patterns were collected with a step rate of 0.02 deg/min, using Cu Kα radiation (λ=1.5418 Å).

- 1. **CdCl_2_ solution preparation.** Cadmium chloride (Sigma, USA) was dissolved in 0.9% medical sterile saline with the final concentration of 0.8 mg/mL. After dissolution, the solution was filtered through 0.22 μm sterile syringe filter (Millipore, Bedford, MA, USA) and stocked in 4℃.
  2. **Plasma biochemical and hematology analysis.** Liver function was evaluated by the plasma level of alanine aminotransferase (ALT) and aspartate aminotransferase (ALT) and total bilirubin (TBIL). Nephrotoxicity was determined by the plasma level of blood urea nitrogen (BUN) and creatinine (Crea). These proteins were analyzed by a biochemical autoanalyzer, (OLYMPUS AU400, Olympus, Tokyo, Japan). The number of red blood cell count (RBC), hemoglobin (HGB), hematocrit (HCT), mean corpuscular volume (MCV), mean corpuscular hemoglobin (MCH), mean corpuscular hemoglobin concentration (MCHC), platelet count (PLT) and white blood cell (WBC) were also determined by an autoanalyzer (ABX 60, France).
  3. **Quantitative gene expression.** Total RNA was extracted from lactating mammary glands using the RNeasy Lipid Tissue Mini Kit (Qiagen, Hilden, Germany) according to the manufacturer’s instructions. cDNA was reverse-transcribed from total RNA (1 μg) using the Transcriptor First Strand cDNA Synthesis Kit (Roche Applied Science, Mannheim, Germany). The cDNA product was amplified by quantitative real-time PCR using the LightCycler® 480 SYBR I Master (Roche Applied Science, Mannheim, Germany). qPCR was performed in the LightCycler® 480 Real-Time PCR System (Roche Diagnostics, Meylan, France). All of the reactions were performed in triplicate. GAPDH was used as an internal control. The primer sequences for the genes were as follows:

| GAPDH | 5’-CCCAGCAAGGACACTGAGCAAGA-3’ (Forward)  5’-ATGGGGGTCTGGGATGGAAATTGT-3’ (Reverse) |
| --- | --- |
| β-casein | 5’-GTCACAGCCCCAGGCCTTTCC-3’ (Forward)  5’-TGGCACCACAGGGGGTTGAG-3’ (Reverse) |
| α- lactalbumin | 5’-GGCTACCTCAGCTTGGCTCTTGC-3’ (Forward)  5’-TTTGCAGTCCAGTGCGCCAGT-3’ (Reverse) |
| EGF | 5’-CCTCGTGCACAGCCCAGGAC-3’ (Forward)  5’-GCATGCGCTCGAGTGGGACT-3’ (Reverse) |
| Lactoferrin | 5’-ACCGAGCGCCTGAAGCAGTG-3’ (Forward)  5’-ACCGTGGCTCCGGGATGAGA-3’ (Reverse) |

The reaction conditions were 95°C for 10 min, followed by 45 cycles of 95°C for 10 s, 55°C for 20 s, and 72°C for 20 s. The results were normalized to GAPDH in the same reaction and are expressed as the fold difference between the experimental and control mice.

- 1. **Milk samples Western blot analysis.** The protein concentrations in the milk samples were determined in a similar manner. Equal volumes of protein (15 μg) were loaded onto 10% SDS/PAGE gels and then electrophoretically transferred onto a polyvinylidene difluoride membrane (Millipore, MA, USA) using a Mini Trans-Blot cell (Bio-Rad, CA, USA). The membranes were then incubated in 5% BSA (Sigma, USA) in TBS with 0.1% Tween 20 and shaken for 1 h at room temperature, followed by incubation overnight at 4°C with primary antibodies against α-lactalbumin (Santa Cruz Biotechnology, Santa Cruz, CA, USA), β-casein (Santa Cruz Biotechnology, Santa Cruz, CA, USA), epidermal growth factor (Millipore, MA, USA), and lactoferrin (Millipore, MA, USA).
