# Supplementary material for: Induction of Size-Dependent Breakdown of Blood-Milk Barrier in Lactating Mice by TiO2 Nanoparticles
Source: PLoS One. 2015 Apr 7;10(4):e0122591. doi: 10.1371/journal.pone.0122591 (PMC4388820; doi:10.1371/journal.pone.0122591)
Supplement: S1 Table — (DOCX) [file pone.0122591.s005.docx]

**S1 Table Blood biochemistry (A) and hematology (B) of dams after TNP-8 and -50 exposure (8 mg/kg) at LD 10.**

A.

|  | **ALT**  **(U/L)** | **AST**  **(U/L)** | **TBIL**  **(μmol/L)** | **BUN**  **(μmol/L)** | **CREA**  **(μmol/L)** |
| --- | --- | --- | --- | --- | --- |
| PBS | 60±8.5 | 108.5±13.1 | 1.46±0.4 | 15.7±1.8 | 15.7±4.0 |
| TNP **1** | 61.2±11.1 | 105.1±8.6 | 1.14±0.2 | 15.5±2.1 | 16.4±1.1 |
| TNP **2** | 61.1±12.3 | 98.4±12.8 | 1.31±0.2 | 15.8±2.0 | 15.9±0.9 |

All data represent mean ± s.d. (n=7).

Note: Alanine aminotransferase (ALT), aspartateaminotransferase (AST), total bilirubin (TBIL), blood urea nitrogen (BUN) and creatinine (CREA).

B.

|  | **RBC**  **(×10^12^/L)** | **HGB**  **(g/L)** | **HCT**  **(%)** | **MCV**  **(fL)** | **MCH**  **(pg)** | **MCHC**  **(g/L)** | **PLT**  **(×10^9^/L)** | **WBC**  **(×10^9^/L)** |
| --- | --- | --- | --- | --- | --- | --- | --- | --- |
| PBS | 9.2±0.4 | 148.9±8 | 45±2.0 | 48.7±1 | 16.2±0.4 | 332.8±6 | 1206.5±189 | 7.9±2 |
| TNP 1 | 9.3±0.5 | 151.1±8 | 45.5±3 | 49.2±1 | 16.3±0.4 | 332.4±4 | 1286.4±158 | 9±3 |
| TNP 2 | 9.0±0.7 | 144.4±9 | 43.8±3 | 48.8±1 | 16.1±0.5 | 330±5 | 1334.4±146 | 9.9±4 |

All data represent mean ± s.d. (n=7).

Note: red blood cell count (RBC), hemoglobin (HGB), hematocrit (HCT), mean corpuscular volume (MCV), mean corpuscular hemoglobin (MCH), mean corpuscular hemoglobin concentration (MCHC), platelet count (PLT) and white blood cell (WBC).
